# Isolation, sequencing, and heterologous expression of the *Paecilomyces variotii* gene encoding *S*-hydroxymethylglutathione dehydrogenase (*fldA*)

**DOI:** 10.1007/s00253-014-6203-8

**Published:** 2014-11-16

**Authors:** Takuji Oka, Yuji Komachi, Kazufumi Ohshima, Yoichi Kawano, Kohsai Fukuda, Kazuhiro Nagahama, Keisuke Ekino, Yoshiyuki Nomura

**Affiliations:** Department of Applied Microbial Technology, Faculty of Biotechnology and Life Science, Sojo University, Ikeda 4-22-1, Kumamoto, 860-0082 Japan

**Keywords:** *Paecilomyces variotii*, Formaldehyde degradation, *S*-hydroxymethylglutathione dehydrogenase, fldA, Heterologous expression

## Abstract

The filamentous fungus *Paecilomyces variotii* NBRC 109023 (teleomorph: *Byssochlamys spectabilis* NBRC 109023) degrades formaldehyde at concentrations as high as 2.4 % (*w*/*v*). In many prokaryotes and in all known eukaryotes, formaldehyde degradation is catalyzed by *S*-hydroxymethylglutathione (*S*-HMGSH) dehydrogenase. We report here the isolation and characterization of the gene encoding *S*-HMGSH dehydrogenase activity in *P. variotii*. The 1.6-kb *fldA* gene contained 5 introns and 6 exons, and the corresponding cDNA was 1143 bp, encoding a 40-kDa protein composed of 380 amino acids. FldA was predicted to have 74.3, 73.7, 68.5, and 67.4 % amino acid identity to the *S*-HMGSH dehydrogenases of *Hansenula polymorpha*, *Candida boidinii*, *Saccharomyces cerevisiae*, and *Kluyveromyces lactis*, respectively. The predicted protein also showed high amino acid similarity (84∼86 %) to the products of putative *fldA* genes from other filamentous fungi, including *Aspergillus* sp. and *Penicillium* sp. Notably, the *P. variotii fldA* gene was able to functionally complement a *Saccharomyces cerevisiae* strain (BY4741 ∆*sfa1*) lacking the gene for *S*-HMGSH dehydrogenase. The heterologous expression construct rendered BY4741 ∆*sfa1* tolerant to exogenous formaldehyde. Although BY4741 (parental wild-type strain) was unable to degrade even low concentrations of formaldehyde, BY4741 ∆*sfa1* harboring *Paecilomyces fldA* was able to degrade 4 mM formaldehyde within 30 h. The findings from this study confirm the essential role of *S*-HMGSH dehydrogenase in detoxifying formaldehyde.

**Electronic supplementary material:**

The online version of this article (doi:10.1007/s00253-014-6203-8) contains supplementary material, which is available to authorized users.

## Introduction

Formaldehyde is a ubiquitous compound that is produced by both biological (Levy 1971; Zimmerman et al. 1978) and environmental sources (Ando 1998). Because formaldehyde displays nonspecific reactivity for proteins and nucleic acids (Grafstrom et al. 1983), it has been used classically in the fixation of biological specimens, preservation (e.g., stabilization of starch-derived adhesives), viscosity stabilization, and disinfection (e.g., room sterilization). Recently, advanced technologies for the generation of potable water have included pretreatment by ozonation, which generates formaldehyde as a result of the reaction of ozone with humic substances (Schechter and Singer 1995). Although the toxic mechanisms of formaldehyde have not been determined in detail, its nonspecific reactivity with biological molecules makes it highly toxic to biological systems. Formaldehyde is also chemically similar to formamide and a number of other chaotropic agents (Cray et al. 2013b) and may therefore induce specific toxic effects, such as chaotropic stress (Hallsworth et al. 2003; Bhaganna et al. 2010). For these reasons, there are increasing concerns about formaldehyde as an environmental pollutant. One approach to reduce environmental pollution is bioremediation. We, therefore, have been screening for microorganisms capable of degrading formaldehyde and isolated a filamentous fungus that degraded formaldehyde at concentrations as high as 2.4 % (*w*/*v*) (0.8 M). The filamentous fungus was identified as *Paecilomyces* sp. No. 5 (Iwahara et al. 2002) and based on subsequent rDNA sequence analysis, it was designated *Paecilomyces variotii* No. 5 (unpublished data). This filamentous fungus has been deposited in the National Institute of Technology and Evaluation (NITE) Biological Resource Center (Kisarazu, Chiba Pref., Japan) as NBRC 109023 (Fukuda et al. 2012).

Formaldehyde-oxidizing enzymes are divided into two groups based on the nature of the electron acceptor, corresponding to NAD(P)^+^-dependent and dye (cytochrome)-linked activities. NAD(P)^+^-dependent formaldehyde-oxidizing enzymes are further subdivided based on the requirement for secondary cofactors, such as thiol compounds, tetrahydrofolate, methylene tetrahydromethanopterin, and modifier proteins (Zahn et al. 2001). The oxidation of formaldehyde in eukaryotic cells is primarily mediated by a NAD^+^- and glutathione-dependent formaldehyde dehydrogenase, designated as *S*-hydroxymethylglutathione (*S*-HMGSH) dehydrogenase (Achkor et al. 2003; Koivusalo et al. 1989). *S*-HMGSH dehydrogenase catalyzes the following reaction:

$$ S-\mathrm{HMGSH}+{\mathrm{NAD}}^{+}\rightleftarrows S-\mathrm{formylglutathione}+\mathrm{NADH}+{\mathrm{H}}^{+} $$

where *S*-HMGSH is a nonenzymatically (Gutheil et al. 1997; Mason et al. 1986; Uotila and Koivusalo 1974) and/or enzymatically formed adduct of glutathione and formaldehyde (Goenrich et al. 2002; Neculai et al. 2005; Wilson et al. 2008). The formation of *S*-HMGSH from formaldehyde and glutathione is a central reaction in the consumption of cytotoxic formaldehyde in many organisms. *S*-HMGSH dehydrogenase is therefore the main enzyme for formaldehyde detoxification in all eukaryotic organisms. The resulting *S*-formylglutathione is further oxidized via formate to carbon dioxide.

We previously reported the purification and characterization of the *S*-HMGSH dehydrogenase from *P. variotii* NBRC 109023 (Fukuda et al. 2012). We report here the isolation and cloning of the *fldA* gene encoding the *P. variotii* NBRC 109023 *S*-HMGSH dehydrogenase. The activity of the gene product was confirmed by heterologous expression and complementation of a *Saccharomyces cerevisiae* strain lacking native *S*-HMGSH dehydrogenase activity.

## Materials and methods

### Microorganisms, media, and culture conditions

*P. variotii* NBRC 109023 (Iwahara et al. 2002; Fukuda et al. 2012) was used as a source of *S*-HMGSH dehydrogenase, RNA, and chromosomal DNA. *S. cerevisiae* BY4741 (*MATa his3*∆*1 leu2*∆*0 met15*∆*0 ura3*∆*0*, parental wild-type strain), and the isogenic mutant ∆*sfa1* (deleted for the *S*-HMGSH dehydrogenase gene) (Wehner et al. 1993) were obtained from EUROSCARF (Frankfurt, Germany) and used for the expression of the NBRC 109023 *fldA* gene. *Escherichia coli* DH5α was obtained from Takara Bio Inc. (Otsu, Japan) and used for plasmid construction and vector propagation.

NBRC 109023 was cultured in minimal medium (MM) consisting of 1 % (*w*/*v*) glucose, 0.6 % (*w*/*v*) NaNO_3_, 0.052 % (*w*/*v*) KCl, 0.052 % (*w*/*v*) MgSO_4_ · 7H_2_O, 0.152 % (*w*/*v*) KH_2_PO_4_, and Hunter’s trace elements, pH 6.5, as described by Barratt et al. (1965); for plates, MM was supplemented with 1.5 % (*w*/*v*) agar. To obtain NBRC 109023 RNA and chromosomal DNA, NBRC 109023 was cultured at 30 °C on solid medium (90-mm petri dish). After 1 week of growth, fungal spores were removed from the plate surface using 5 ml sterilized water. The resulting spore suspension was used to inoculate 100 ml MM liquid medium in a 500-ml shake flask, which was then incubated aerobically with shaking (127 strokes/min) at 30 °C. After 24 h, formaldehyde was added to culture at a final concentration of 0.3 % (*w*/*v*), and the culture was further incubated with aerobic shaking for 4 h to induce expression of genes related to formaldehyde metabolism. The resulting mycelial suspension was harvested by filtration using gauze, and the mycelial pellets were used immediately for preparing RNA or stored at −30 °C until used for DNA preparation.

*E. coli* DH5α was cultured in Luria-Bertani (LB) medium (2 % (*w*/*v*) Bacto Tryptone, 0.5 % (*w*/*v*) dried yeast extract, and 0.5 % (*w*/*v*) NaCl). When necessary, plasmid selection was maintained by supplementation of the medium with 50 μg/ml ampicillin.

*S. cerevisiae* was cultured in a synthetic minimal (SD) liquid medium composed of 0.67 % (*w*/*v*) yeast nitrogen base without amino acids (Difco Laboratories Inc., Detroit, MI, USA) and 0.5 % (*w*/*v*) d-glucose (Wako Pure Chemical Industries, Osaka, Japan) and supplemented with an amino acid mixture and 1.5 % (*w*/*v*) agar (for solid medium). Formaldehyde tolerance was tested by serial dilution and spotting on SD (-Ura) plates containing the indicated concentration of formaldehyde. Quantitative spot-test assays are successfully used in diverse types of study and for a range of microbial species (Matecic et al. 2010; Chin et al. 2010; Cray et al. 2013a; Pais et al. 2013; Baerends et al. 2008). We also performed assays to confirm the tolerance of the transformant harboring YEp352GAPII-fldA to formaldehyde. The spot-test assay was performed using BY4741 or BY4741 ∆*sfa1* mutant transformed with the empty vector YEp352GAPII (Nakayama et al. 2003) containing the *S. cerevisiae* glyceraldehyde-3-phosphate dehydrogenase (GAP) promoter and terminator, or BY4741 ∆*sfa1* mutant containing the NBRC 109023 *fldA* gene (YEp352GAPII-fldA). Yeast liquid cultures were grown aerobically at 30 °C in SD (-Ura) for 24 h, and the cell suspensions were then serially diluted to the indicated cell densities (Fig. 4) and spotted on SD (-Ura) agar supplemented with formaldehyde (0–8 mM). The plates were incubated at 30 °C for 2 days, and cell growth was evaluated. Formaldehyde degradation by these yeast strains was also investigated in liquid culture in the presence of formaldehyde. Briefly, overnight seed cultures of *Saccharomyces* were grown at 30 °C in SD (-Ura) liquid medium, and 40 μl of the seed culture was used to inoculate 40-ml liquid cultures of SD (-Ura) containing 2 or 4 mM formaldehyde. The concentration of formaldehyde in the cultures was determined using a formaldehyde test kit (Wako Pure Chemical Industries). Growth was determined based on frequent measurement of optical density at 600 nm.

### Determination of amino acid sequences of peptide fragments

*S*-HMGSH dehydrogenase of NBRC 109023 was purified as reported previously (Fukuda et al. 2012). The purified enzyme was *S*-pyridylethylated as previously described (Friedman et al. 1970). Briefly, 5 mg of purified *S*-HMGSH dehydrogenase was dissolved in 900 μl of 0.5 M Tris-HCl buffer (pH 8.5) containing 7 M guanidine HCl and 10 mM ethylenediaminetetraacetic acid (EDTA). After the air phase was replaced with nitrogen gas, 3 μl of 4-vinylpyridine was added to the buffer solution, which was then mixed well before 6 μl of tributyl-phosphine was added to the mixture. The reaction was carried out overnight in darkness under a nitrogen gas atmosphere. The reaction mixture was then dialyzed against distilled water and lyophilized. The obtained *S*-pyridylethylated enzyme was digested with *Staphylococcus aureus* protease (V8 protease) or *Achromobacter* lysyl endopeptidase (Takara Bio Inc.) at 37 °C for 12 h. The resulting peptide mixtures were separated by reverse phase high-performance liquid chromatography (HPLC) on a TSKgel ODS-120 T column (C18, 4.6 × 250 mm; Tosoh Co., Osaka, Japan). The HPLC separation was performed at a rate of 1.0 ml/min with 0.1 % (*v*/*v*) trifluoroacetic acid (TFA) in water as solvent A and 0.1 % (*v*/*v*) TFA in 60 % (*v*/*v*) acetonitrile as solvent B (mixture with ratio of 2:3 of solvent A and acetonitrile). The column was first equilibrated with solvent A, and the ratio of solvent B was then increased to 20 % (*v*/*v*) for 5 min. Peptide fragments were eluted by a linear gradient of solvent B (from 20 % (*v*/*v*) to 100 % (*v*/*v*)) over 55 min. Separated peptide fragments were collected and dried using a centrifugal concentrator (TOMY concentrator CC-180; Tomy Seiko Co., Ltd., Tokyo, Japan). The dried peptide fragments were dissolved in 20 μl of a 30 % (*v*/*v*) acetic acid solution and applied to a Protein Sequencer (model 476; Applied Biosystems Inc., Foster, CA, USA) to determine the amino acid sequences.

### Design of PCR primers

The PCR primers used in this study are listed in Table 1. P1-FW and P1-RV were designed based on the partial amino acid sequence of *S*-HMGSH dehydrogenase from NBRC 109023. FldA-RT-FW and FldA-RT-RV were designed based on the DNA sequence of the *fldA* gene.

**Table 1:** PCR primers used in this study

Primer	Nucleotide sequence (5′-3′)
P1-FW	GC(A/T/G/C)GC(A/T/G/C)GT(A/T/G/C)GC(A/T/G/C)TGGGC
P1-RV	(A/G)CA(T/C)TC(A/T/G/C)CC(A/G)CA(T/C)TC(A/T/G/C)GG
FldA-RT-FW	AGAATTCATGCATCACCATCACCATCACGCCAGCACTGTCGGTAAAAC
FldA-RT-RV	AAAAACTCGAGTTAAGCCTTCATATCCAAAACACAGCG

### Isolation of the *fldA* gene

Total RNA was prepared using TRIzol RNA Isolation Reagent (Life Technologies Corp., Carlsbad, CA, USA) according to the supplier’s instructions. mRNA was purified using an Oligotex-dT30 <super> mRNA purification kit (from total RNA) (Takara Bio Inc.) according to the supplier’s instructions. A partial 260-bp DNA fragment of the *S*-HMGSH dehydrogenase gene of NBRC 109023 was synthesized by PCR using cDNA as template and primers P1-FW and P1-RW and was then cloned into pGEM-5Zf (+) using the TA cloning method with the pGEM-T Easy Vector System (Promega Co., Madison, WI, USA) to yield pGEM-pfldA. A partial fragment (260 bp) of the *fldA* gene was amplified by PCR with primers P1-FW and P1-RV and the plasmid pGME-pfldA as a template. The resultant DNA fragment was labeled using a Digoxigenin (DIG) DNA Labeling Kit (Roche Diagnostics, Basel, Switzerland) and used as a DNA probe for colony hybridization to screen for cosmids containing the complete *S*-HMGSH dehydrogenase gene of NBRC 109023. Chromosomal DNA was extracted according to the modified method described by Raeder and Broda (1985). Briefly, NBRC 109023 was cultured in MM liquid medium, and mycelia were then collected by filtration using gauze and washed twice with sterilized water. Washed mycelia were stored at −30 °C until use. Frozen mycelia were placed in liquid nitrogen and then ground into a powder using a mortar and pestle. Powdered cells were resuspended in extraction buffer (200 mM Tris-HCl (pH 8.5), 250 mM NaCl, 25 mM EDTA, and 0.5 % (*w*/*v*) sodium dodecyl sulfate (SDS)) and then subjected twice to phenol/chloroform treatment. The obtained supernatant was dialyzed with distilled water. A genomic DNA library of NBRC 109023 was constructed as follows: genomic DNA was partially digested with *Sau*3AI (Nippon Gene Co., Ltd., Tokyo, Japan) and the generated 10 to 25-kbp DNA fragments were fractionated. The obtained DNA fragments were inserted randomly into the cosmid vector Charomid 9-28 (Saito and Stark 1986) after digestion with *Bam*HI and were then subjected to in vitro packaging using a LAMBDA INN in vitro packaging kit (Nippon Gene). *E. coli* (XL1-Blue MRF) transformants carrying the *fldA* gene of NBRC 109023 were analyzed by colony hybridization using a 260-bp DIG-labeled probe. One positive colony was isolated, and the nucleotide sequence of the *fldA* gene region (2319 bp) in the cosmid clone was determined by the primer-walking method. DNA sequences of the isolated cosmids were analyzed using an ABI PRISM 3100 Genetic Analyzer (Applied Biosystems). Oligonucleotide synthesis was performed by Hokkaido System Science Co., Ltd. (Sapporo, Japan).

### Isolation of cDNA of the *fldA* gene and construction of a yeast expression plasmid for the *fldA* gene

To isolate cDNA of the *fldA* gene, RT-PCR was carried out with an Access Quick RT-PCR system (Promega Co.) using purified mRNA as template and the primers FldA-RT-FW, which contained an *Eco*RI site upstream of the initiation codon and a coding sequence for a histidine tag downstream of the initiation codon, and FldA-RT-RV, which contained a *Xho*I site downstream of the stop codon (Table 1). The obtained PCR product was subjected to agarose electrophoresis. The cDNA product was extracted from the gel using the QIAquick Gel Extraction Kit (Qiagen, Hilden, Germany) and was then cloned using the pGEM-T Easy Vector System (Promega Co.) to yield pGEM-fldA. Amplified pGEM-fldA was digested with *Eco*RI and *Xho*I, and the resultant 1.2-kb DNA fragments were inserted into the *Eco*RI and *Sal*I sites in YEp352GAPII to yield YEp352GAPII-fldA. The DNA sequence of YEp352GAPII-fldA was confirmed by sequence analysis on an ABI PRISM 3100 Genetic Analyzer (Applied Biosystems).

### Construction of recombinant *S. cerevisiae* strains

Transformation of *S. cerevisiae* was performed by the lithium acetate method (Klebe et al. 1983). After transformation, a diluted cell suspension was plated on SD (-Ura) agar medium and incubated at 30 °C for 3 days. This method was used to construct strains SU1, *S. cerevisiae* BY4741 harboring YEp352GAPII, SU2, BY4741 (∆*sfa1*) harboring YEp352GAPII, and SU3, BY4741 (∆*sfa1*) harboring YEp352GAPII-fldA.

### Western blot analysis of His-tagged *S*-HMGSH dehydrogenase proteins

Strains SU1, SU2, and SU3 were cultured aerobically at 30 °C for 24 h. Cells were washed three times with sterilized water and were then disrupted using Y-PER Yeast Protein Extraction Reagent (Thermo Fisher Scientific, Waltham, MA, USA). After removing cell debris by centrifugation at 21,000×*g* for 30 min, proteins in the supernatants were analyzed by Western blotting. For the analysis, proteins were separated by SDS polyacrylamide gel electrophoresis with a 10 % (*w*/*v*) gel according to the method of Laemmli (1970) and were then transferred to a polyvinylidene difluoride membrane. His-tagged protein was detected with Anti-Penta-His antibody (Qiagen) and goat anti-mouse IgG-HRP (Santa Cruz Biotechnology, Inc., Dallas, TX, USA). An ECL Prime Western Blotting Detection System (GE Healthcare, Little Chalfont, UK) was used to visualize immunoreactive proteins. Chemical fluorescent signals on the membrane were recorded using a MicroChemi imaging system (Berthold Technologies, Bad Wildbad, Germany). Imaging of the ELC-treated membranes was stopped before saturation of the signals.

### Nucleotide sequence accession number

The nucleotide sequence of *fldA* was deposited in the EMBL/GenBank/DDBJ databases under the accession number AB871646. Protein sequences for phylogenetic analysis were obtained from the following databases: *Aspergillus* species, http://www.aspergillusgenome.org/; *S. cerevisiae*, http://www.yeastgenome.org/; and for all the other species, http://www.ncbi.nlm.nih.gov/. The accession numbers of the protein sequences are as follows: Afu2g01040 (*A. fumigatus* Af293), An10g00510 (*A. niger* CBS513.88), AN7632 (*A. nidulans* FGSC4), AO090308000002 (*A. oryzae* RIB40), X82647 (*Arabidopsis thaliana*), NP_001105485 (*Zea mays*), Os02g0815500 (*Oryza sativa* Japonica group), CAG38730 (*Homo sapiens*), AAH90978 (*Mus musculus*), XM_007630816 (*Cricetulus griseus*), NP_571924 (*Danio rerio*), KLLA0D12342g (*Kluyveromyces lactis*), and YDL168W (*Saccharomyces cerevisiae*). For the construction of a phylogenetic tree with sequences related to FldA, Clustal W software was used (Thompson et al. 1994).

## Results

### Determination of the partial amino acid sequence of *S*-HMGSH dehydrogenase from NBRC 109023

**Fig. 1:**
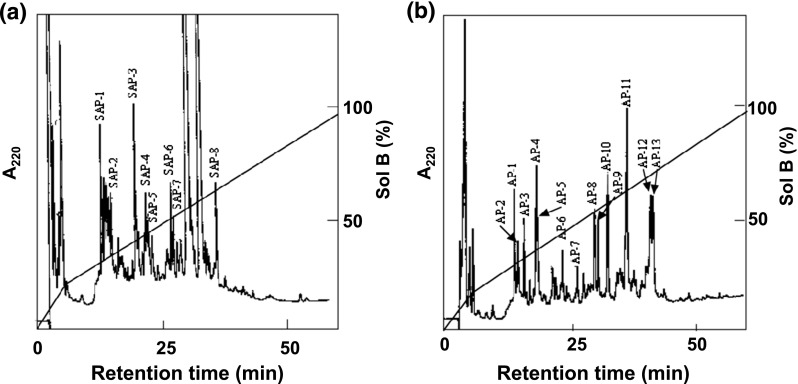
HPLC separation profiles of peptide fragments produced by protease digestion of *S*-HMGSH dehydrogenase from *Paecilomyces variotii* NBRC 109023. **a** Digestion by *Staphylococcus aureus* protease and **b** digestion by *Achromobacter* protease. HPLC separation of the resulting peptide fragments was performed at a rate of 1.0 ml/min with solvent A (0.1 % (*v*/*v*) TFA in water) and solvent B (0.1 % (*v*/*v*) TFA in 60 % (*v*/*v*) acetonitrile). A TSK gel ODS-120T column was used for the separation. The column was equilibrated with solvent A, and peptide fragments were eluted by a linear gradient of solvent B (from 20 % (*v*/*v*) to 100 % (*v*/*v*)) over 55 min

As reported previously (Fukuda et al. 2012), initial attempts to determine the amino acid sequence of the *S*-HMGSH dehydrogenase from NBRC 109023 were frustrated by an N-terminal domain that prevented Edman degradation. To overcome this difficulty, here, the purified enzyme was *S*-pyridylethylated and cleaved with two types of proteases. HPLC separation patterns of the obtained peptide fragments are shown in Fig. 1. The amino acid sequences of the peptide fragments were determined, and the sequences of five selected peptide fragments (SAP-1, AP-4, AP-5 AP-7, and AP-8) are shown in Table 2. The amino acid sequences of the HPLC-separated peptides were used to generate the protein sequence of the S-HMGSH dehydrogenase from NBRC 109023 by reference to the sequence of the *S*-HMGSH dehydrogenase from *Candida boidinii* (Lee et al. 2002). The determined 91 amino acid sequence of the *S*-HMGSH dehydrogenase from NBRC 109023 is shown in Table 3.

**Table 2:** Amino acid sequences of peptide fragments obtained by enzymatic digestion of *S*-HMGSH dehydrogenase

Fragment	Amino acid sequence
SAP-4	NH_2_-AYTLSGLDP-COOH
AP-4	NH_2_-PGDRVIALYTPECGEC-COOH
AP-5	NH_2_-AHEVRIQIIHTGVCHTDAY-COOH
AP-7	NH_2_-AAVAWAAGEPFSVEDVQVAPPK-COOH
AP-8	NH_2_-DPEGDFPVIKGHEGAGIVESVGEGVTQVK-COOH

**Table 3:** Partial amino acid sequence of *S*-HMGSH dehydrogenase

NH_2_-AAVAWAAGEPFSVEDVQVAPPKAHEVRIQIIHTGVCHTDAYTLSGL
DPEGDFPVIKGHEGAGIVESVGEGVTQVKPGDRVIALYTPECGEC-COOH

### Isolation and DNA sequence of the *fldA* gene in the genome of NBRC 109023

**Fig. 2:**
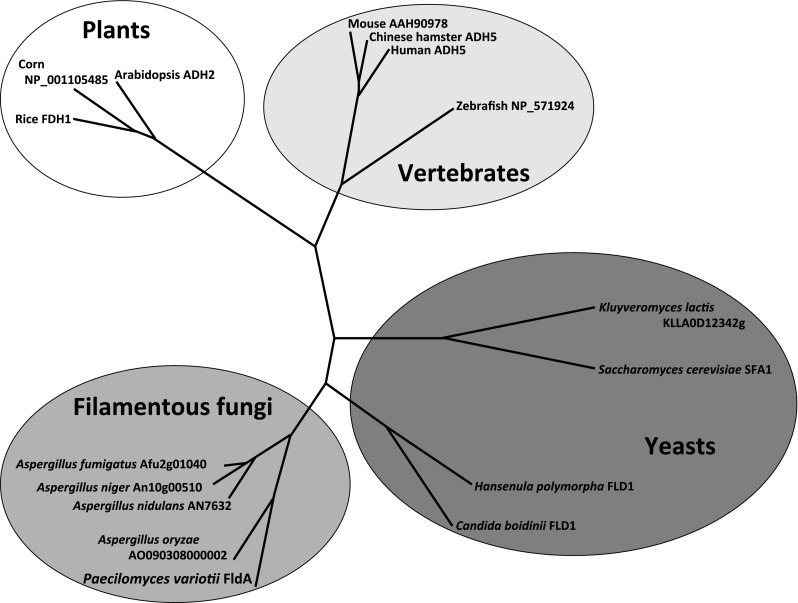
Phylogenetic tree of *S*-HMGSH dehydrogenases from *A. fumigatus* Af293 (Afu2g01040), *A. niger* CBS513.88 (An10g00510), *A. nidulans* FGSC4 (AN7632), *A. oryzae * RIB40 (AO090308000002), *Arabidopsis thaliana* (ADH2), *Zea mays* (NP_001105485), *Oryza sativa* Japonica group (Os02g0815500), *Homo sapiens* (CAG38730), *Mus musculus* (AAH90978), *Cricetulus griseus* (XM_007630816), *Danio rerio* (NP_571924), *Kluyveromyces lactis* (KLLA0D12342g), and *Saccharomyces cerevisiae* (YDL168W). Clustal W was used for multiple sequence alignments

*E. coli* (XL1-Blue MRF) transformants carrying genomic DNA encoding the entire *fldA* gene were screened by colony hybridization. We detected potential initiation and stop codons in the fragment containing *fldA* gene of NBRC 109023. Analysis of the complete cDNA sequence revealed that the *fldA* gene was 1143 bp, encoding a protein of 380 amino acids. From these results, it was confirmed that the *fldA* gene is 1614 bp in length in the NBRC 109023 genome and is composed of 5 introns and 6 exons (Fig. S1; EMBL/GenBank/DDBJ accession number AB871646). The 5′ and 3′ splice joints of all introns of *fldA* contained GU and AG sequences, indicating that the introns conformed to the GU/AG rules proposed by Breathnach et al. (1978). Phylogenetic analysis indicated that FldA proteins are widely distributed from yeasts to humans and that the FldA proteins of filamentous fungi are distinct from those of yeast (Fig. 2).

### Heterologous expression of NBRC 109023 *fldA*

**Fig. 3:**
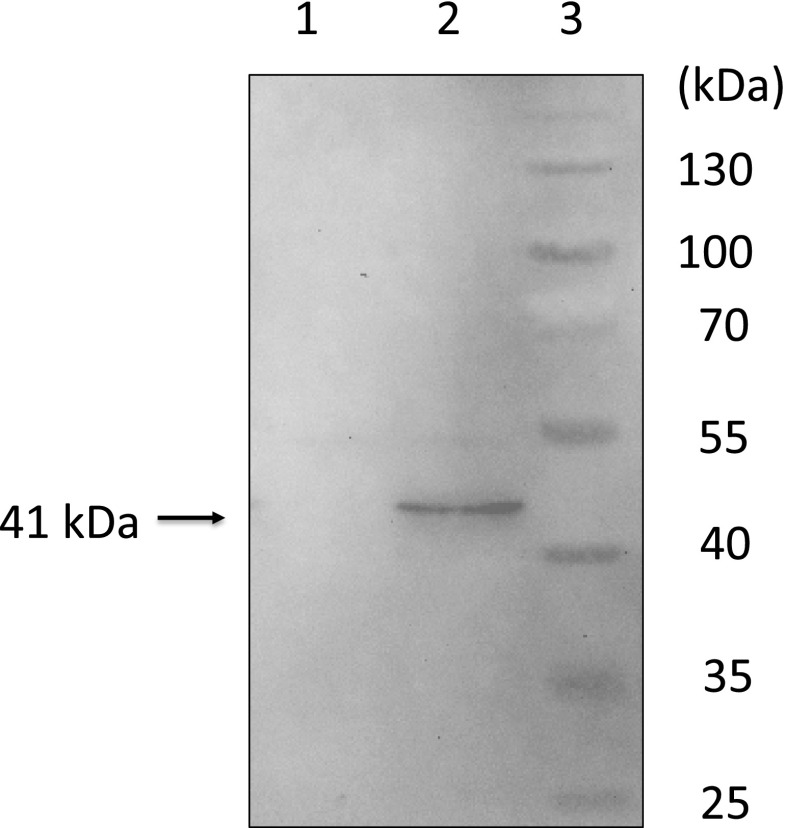
Western blot analysis of S-HMGSH dehydrogenase in *S. cerevisiae* (∆*sfa1*) transformed with YEp352GAPII (SU2 strain) and *S. cerevisiae* (∆*sfa1*) transformed with YEp352GAPII-fldA (SU3 strain). Cells were cultured in SD (-Ura), collected, and then disrupted. After cell disruption, cell debris was removed by centrifugation, and the supernatant was used as crude enzyme solution. The crude enzyme solutions were subjected to SDS-PAGE and Western blot analysis with Anti-Penta-His antibody and goat anti-mouse IgG antibody. *Lane 1*: *S. cerevisiae* BY4741 (∆*sfa1*) transformed with empty vector YEp352GAPII (SU2 strain). *Lane 2*: *S. cerevisiae* BY4741 (∆*sfa1*) transformant, *fldA* expression strain (SU3 strain). *Lane 3*: protein marker

To confirm the nature of the *fldA* gene product, the ORF was amplified to include a sequence coding for an N-terminal 6-His tag. The gene was cloned under control of the *S. cerevisiae GAP* promoter, and the expression plasmid was transformed into *S. cerevisiae*. Immunoblotting and probing the lysates of the transformed yeast cells with an anti-His-tag antibody revealed the presence of a novel 41-kDa protein (Fig. 3), which is consistent with the predicted size of the His-tagged fusion protein.

### Functional characterization of the NBRC 109023 *fldA* gene product

**Fig. 4:**
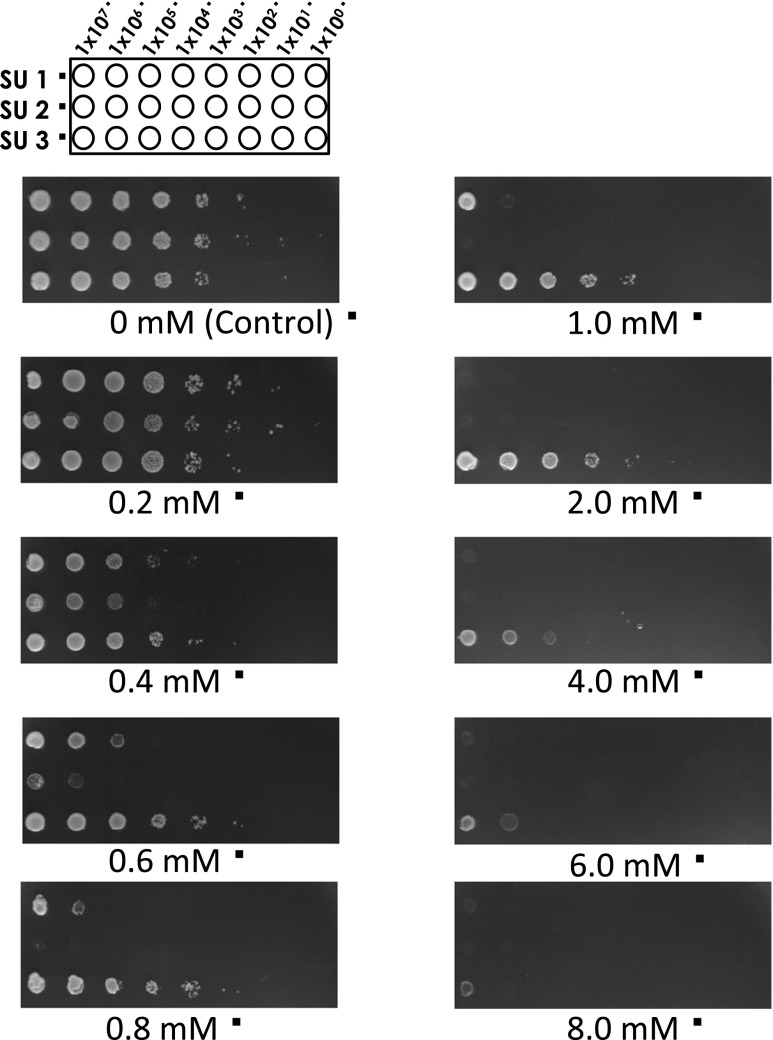
Growth of *S. cerevisiae* strains SU1, SU2, and SU3 on SD (-Ura) agar plates containing 0 to 8.0 mM formaldehyde. Cell suspensions were serially diluted at the indicated concentrations and then spotted on SD (-Ura) agar plates. The plates were incubated at 30 °C for 2 days, and growth was then evaluated. *SU1*: *S. cerevisiae* BY4741 transformed with empty vector (YEp352GAPII). *SU2*: *S. cerevisiae* BY4741 (∆*sfa1*) transformed with empty vector (YEp352GAPII). *SU3*: *S. cerevisiae* BY4741 (∆*sfa1*) transformed with YEp352GAPII-fldA

We next investigated whether heterologous expression of NBRC 109023 *fldA* would endow a host yeast strain lacking an endogenous gene encoding S-HMGSH dehydrogenase (*sfa1*) with tolerance to formaldehyde (Fig. 4). Growth of SU1, a *SFA1* ^+^ strain harboring the empty vector, was impaired in the presence of 1.0 mM formaldehyde. Growth of SU2, a ∆*sfa1* strain harboring the empty vector, was also completely inhibited by 0.8 mM formaldehyde. In contrast, SU3 (∆*sfa1* transformed with the NBRC 109023 *fldA* expression plasmid) was able to grow in the presence of formaldehyde at concentrations of up to 8.0 mM. Thus, expression of the NBRC 109023 *fldA* gene in a formaldehyde-sensitive yeast strain imparted tolerance to formaldehyde.

### Formaldehyde degradation by SU3

**Fig. 5:**
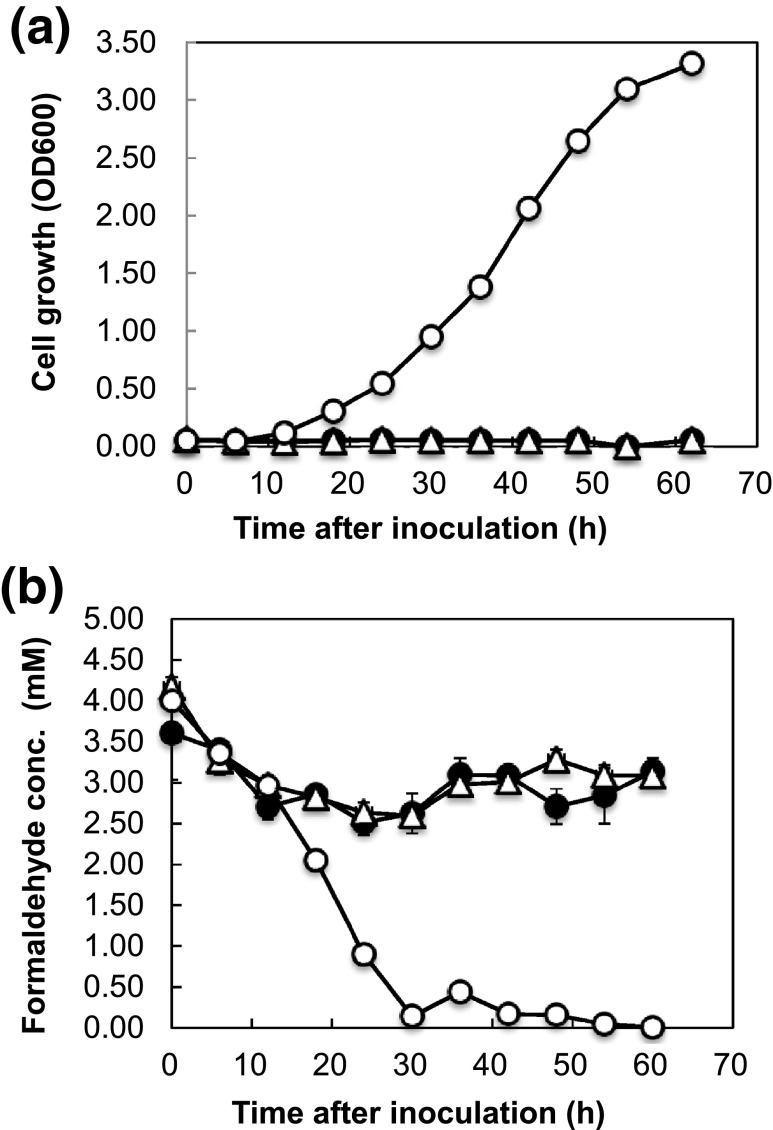
Time courses of formaldehyde degradation by *S. cerevisiae* expressing the *Paecilomyces* S-HMGSH gene. **a** Cell growth and **b** degradation of formaldehyde. *S. cerevisiae* strains were cultured aerobically in SD (-Ura) liquid medium containing 4 mM formaldehyde at 30 °C for 60 h. The *S. cerevisiae* strains used in this experiment were the same as those used in Fig. 4. Symbols: *open circle*, SU3; *triangle*, SU2; *closed circle*, SU1. The *error bars* represent standard deviation (*n* = 3)

To explore the degradation dynamics of formaldehyde, *Saccharomyces* strains were cultured in SD (-Ura) medium containing 2 mM (data not shown) or 4 mM formaldehyde. The time courses of formaldehyde degradation by the yeast strains SU1, SU2, and SU3 are shown in Fig. 5. SU1 and SU2 were unable to grow in the presence of 2 mM formaldehyde. In contrast, SU3 was able to grow in the presence of 4 mM formaldehyde after a long lag period. Although the growth of SU3 appeared to be suppressed due to the toxicity of 4 mM formaldehyde at the beginning of the cultivation period after 20 h, SU3 grew exponentially and the formaldehyde was degraded linearly within 30 h. In medium supplemented with 2 mM formaldehyde, SU3 completely degraded the formaldehyde in 24 h (data not shown). Based on these results, NBRC 109023 *fldA* was able to functionally complement the yeast *sfa1* mutant, confirming that *fldA* encodes a *S*-HMGSH dehydrogenase.

## Discussion

Formaldehyde is a toxic compound that is produced from biological and environmental sources. In many organisms, the first reaction in the degradation of formaldehyde is the formation of *S*-HMGSH from formaldehyde and glutathione. Biochemical and genetic studies in several eukaryotes indicate that the main enzyme responsible for the degradation of formaldehyde is a NAD^+^- and glutathione-dependent formaldehyde dehydrogenase (*S*-HMGSH dehydrogenase). In previous work, we isolated a strain of *P. variotii* NBRC 109023 that degraded formaldehyde at concentrations as high as 2.4 % (*w*/*v*) (Iwahara et al. 2002) and purified the corresponding *S*-HMGSH dehydrogenase responsible for the activity (Fukuda et al. 2012). As we noted previously, sequencing of the protein was complicated by a modification of the N-terminus that precluded Edman degradation. In the present work, we therefore used an alternative proteolytic approach to obtain proteolytic fragments for sequencing, and used the resulting amino acid sequences to identify the *fldA* gene in the *P. variotii* NBRC 109023 genome.

To determine why *P. variotii* NBRC 109023 has a high tolerance to formaldehyde, we analyzed the DNA sequence of *S*-HMGSH dehydrogenase. The full-length cDNA of *fldA* is 1143 bp and encodes a predicted protein of 380 amino acids; the genomic DNA is transcribed and processed from a 1.6-kb region as mRNA containing 5 introns and 6 exons.

We analyzed the amino acid composition of the *S*-HMGSH dehydrogenase of NBRC 109023 from cDNA using GENETYX software and confirmed that this enzyme is composed of 380 amino acids. Specifically, the sequence contained 194 hydrophobic (51.05 %), 98 hydrophilic (25.79 %,) and 88 neutral amino acids (23.16 %), suggesting that this recombinant enzyme is highly hydrophobic. Protein homology searches revealed that the NBRC 109023 FldA protein is homologous to other fungal *S*-HMGSH dehydrogenases, with 74.3, 73.7, 68.5, and 67.4 % identity to enzymes from *Hansenula polymorpha*, *Candida boidinii*, *S. cerevisiae*, and *Kluyveromyces lactis*, respectively. The FldA protein also showed high similarity (84∼86 %) to products of putative *fldA* genes from other filamentous fungi, including those of *Aspergillus* sp. and *Penicillium* sp. To our knowledge, however, there is no report that these filamentous fungi are able to degrade formaldehyde and these homologous proteins have not been characterized.

Heterologous expression of a His-tagged variant of FldA in yeast confirmed that *fldA* from NBRC 109023 encodes a protein of ∼41 kDa. We previously estimated (by SDS-PAGE) that this enzyme has a molecular weight of 49 kDa (Fukuda et al. 2012). This difference in size may reflect posttranslational modification, such as phosphorylation, N-acetylation, and/or N-acylation, of the endogenous *S*-HMGSH dehydrogenase in *Paecilomyces*. However, no such modifications were apparent upon heterologous expression of the NBRC 109023 gene in yeast, perhaps due to differences in substrate specificities of protein modification enzymes or the influence of the 6×His N-terminal tag. Consistent with this speculation, the N-terminus of *S*-HMGSH dehydrogenase purified from NBRC 109023 is blocked against Edman degradation (Fukuda et al. 2012), indicating that the N-terminus is modified.

We investigated whether the heterologous expression of *fldA* in yeast yielded increased tolerance to formaldehyde. SU1, a *SFA*1^+^ strain harboring an empty vector, grew poorly at formaldehyde concentrations up to 1.0 mM. The isogenic SU2 strain, which also harbored an empty vector, but lacked the gene for endogenous (yeast) *S*-HMGSH dehydrogenase, was unable to grow at formaldehyde concentrations exceeding 0.8 mM. In contrast, SU3 (∆*sfa1* carrying a *fldA*-encoding plasmid) was able to grow in the presence of formaldehyde concentrations of up to 8.0 mM. We also investigated the degradation of 2 and 4 mM formaldehyde by SU1, SU2, and SU3 strains. Neither SU1 nor SU2 were able to grow or degrade formaldehyde, indicating that these strains are unable to degrade formaldehyde at these concentrations due to toxicity. In contrast, SU3 harboring the NBRC 109023 *fldA* gene was able to grow in the presence of and completely degrade 2 and 4 mM formaldehyde in 24 and 30 h, respectively. These experimental findings demonstrate that the NBRC 109023 *fldA* gene was functionally expressed in the heterologous yeast host.

No studies have examined heterologous expression of *S*-HMGSH dehydrogenases from filamentous fungi in *S. cerevisiae*, and most studies have been limited to enzymes from *Arabidopsis* (Achkor et al. 2003) and *Hansenula polymorpha* (Baerends et al. 2008). Additionally, heterologous expression of the *S*-HMGSH dehydrogenase genes of maize (Fliegmann and Sandermann 1997) and *Brevibacillus brevis* (Nian et al. 2013) has been reported in *E. coli* and tobacco, respectively. To our knowledge, the present report represents the first identification and characterization of a *S*-HMGSH dehydrogenase gene from a filamentous fungus.

Recently, we reported the draft genome sequence for *P. variotii* NBRC 109023 (Oka et al. 2014), which was found to contain a gene encoding a second putative *S*-HMGSH dehydrogenase. We are currently investigating the possible role of this gene product in formaldehyde tolerance in *P. variotii* NBRC 109023.

## Electronic supplementary material

Below is the link to the electronic supplementary material.

ESM 1 (PDF 134 kb)
