# Supplementary material for: Isolation, sequencing, and heterologous expression of the Paecilomyces variotii gene encoding S-hydroxymethylglutathione dehydrogenase (fldA)
Source: Appl Microbiol Biotechnol. 2014 Nov 16;99(4):1755–63. doi: 10.1007/s00253-014-6203-8 (PMC4322224; doi:10.1007/s00253-014-6203-8)
Supplement: Supplementary file 1 — (PDF 134 kb) [file 253_2014_6203_MOESM1_ESM.pdf]

Supplementary material

Journal name: Applied Microbiology and Biotechnology

Article title: Isolation, sequencing, and heterologous expression of the *Paecilomyces variotii* gene encoding *S*-hydroxymethylglutathione dehydrogenase (*fldA*)

Takuji Oka, Yuji Komachi, Kazufumi Ohshima, Yoichi Kawano, Kohsai Fukuda, Kazuhiro Nagahama, Keisuke Ekino and Yoshiyuki Nomura*

Department of Applied Microbial Technology, Faculty of Biotechnology and Life Science, Sojo University, Ikeda 4-22-1, Kumamoto 860-0082, Japan.

* Corresponding author
Yoshiyuki Nomura
E-mail address: nomura@bio.sojo-u.ac.jp

5′– ggaggcacgtttacgagctatcggattcttggcgcgggacgacatgtgaagtggaagtttgaagctgtattattggatgagtagtctacctaatacagtccccgacgtaccgtaaatccattga
gcggggtaaagccgtaaagtttaaattgaagctttatcattggattattactccttcccataacaagtgtgtcctagactctccaagcatcagaaagggaaaaaacctaagctacacggtagat
ttgaaacccctcactgcttagcatgcggagactggaatgattgagtcatattttcgtaggagatacacgatagaacgtaggcctttggagtctgagcgttggtaaaacattggttgaccgtctc
cacatgtacctacaccaggtcacatcccgccatggaagatgaaattgatcattaatcggaccctcctgaaagcttcttcattattcactgggcactgatgactcgttgaaaccgtaaacgtcat
tgttttcgctcaagctctccgcgataagagcccacgttcgatacgcacaggaggggctgcactatataaccgcgatatctcgatccgtacaagttgtcttttctaattctatatcgacacttca
tcattcacatctatcctccaatattgtcgacacagaaaat

ATGGCCAGCACTGTCGGTAAAgtgagtttcctgcggacttccccccctccccttcctcttggggtcctttctttcattccaaatattcccccattcaagta
M A S T V G K
intron 1

gaggacttcacatcaccacaggactcctcttgcaactggttatatagcatgatcttgtgttatcatatcgttgagtctcagctaacattaagcaatagACT
T

ATCACCTGCAAGgtacgaatgtccccgacatattcctagcgatccttggcttttatctaccaactgagaacttgatggctgatattaccagGCCGCTGTCG
I T C K
intron 2
A A V

CATGGGCTGCCGGCGAGCCATTTTCTGTCGAAGATGTCCAGGTTGCTCCCCCGAAGGCCCACGAGGTTCGCATTCAGATTATCCACACAGGCGTCTGCCAC
A W A A G E P F S V E D V Q V A P P K A H E V R I Q I I H T G V C H

ACCGgttcgtttgaccttttcgagattgaatcccaaagtccgtggttttcttgtctgggttcaaattgtctgacatcgacaattagACGCATATACCCTTT
T
intron 3
D A Y T L

CTGGGAAGGACCCTGAGGGAGATTTCCCTGTTATCCTTGGCCATGAGGGCGCAGGTATTGTTGAGTCGGTCGGTGAAGGCGTGACTACCGTCAAGCCTGGT
S G K D P E G D F P V I L G H E G A G I V E S V G E G V T T V K P G

GACCGGGTGATTGCTCTCTAgtaagatgctctgtcgcattacacctaatacgaatattggctaattgttacaagCACCCCTGAATGTGGTGAATGTAAATT
D R V I A L Y
intron 4
T P E C G E C K F

CTGCAAGTCAGGAAAGACCAACCTGTGCCAGAAGATTCGCGCCACCCAGGGTAAAGGGCTGATGCCCGACGGAACCACCCGCTTCAAGGCGCGCGGAAAGG
C K S G K T N L C Q K I R A T Q G K G L M P D G T T R F K A R G K

ACATATTGCATTATATGGGAACTTCCACCTTTTCCCAATACACTGTTGTGGCAGACATCTCTGTTGTGGCTATTACTGAGAAAGCTCCAACTGATCGAGCA
D I L H Y M G T S T F S Q Y T V V A D I S V V A I T E K A P T D R A

TGTCTATTGGGCTGTGGAATTACCACCGGCTATGGCGCCGCAACTGTTACCGCCAATGTTGAAAAAGGTTCTAACGTCGCTGTCTTTGGTGCTGGATGCAT
C L L G C G I T T G Y G A A T V T A N V E K G S N V A V F G A G C I

TGGTTTGTCTGTTATTGAGGGTGCAGTCAAGAATAAAGCCCACAAAATCATCGTAGTCGACGTGAACGATGACAAGGAAGCATGGGCTCGCAAATTCGGCG
G L S V I E G A V K N K A H K I I V V D V N D D K E A W A R K F G

CCACGGACTTCGTGAACCCTACTAAATTAGGTAACAAGACTGTCCAAGAACAGCTTATCGAGATGACCGATGGTGGGTGCGATTATACTTTCGACTGTACT
A T D F V N P T K L G N K T V Q E Q L I E M T D G G C D Y T F D C T

GGAAATGTCGGTGTTATGCGTACTGCTCTTGAATCTTGCCATAAAGGTTGGGGCCAAAGCATCATCATTGGCGTCGCCGCGGCCGGTCAAGAAATCTCTAC
G N V G V M R T A L E S C H K G W G Q S I I I G V A A A G Q E I S T

AAGACgtaagtttgttcccgccgtgttcccaccgttgttggcagcttactttatgcaattgctgttgctcactgggtcattagCATTCCAACTAGTTACGG
R
intron 5
P F Q L V T

GGCGTGTGTGGAAAGGCTGTGCGTTCGGTGGCATCAAAAGTCGCTCCCAACTACCTAGTCTTGTCGAAGACTATGTTACCGGCTTCCTTAGGGTTGATGAT
G R V W K G C A F G G I K S R S Q L P S L V E D Y V T G F L R V D D

TATATTACTCATCGTGAATCTCTGGGCACCATCAACACTGCTTTCAAGCATATGAAGAGTGGTGACTGTATCCGCTGTGTTTTGGATATGAAGGCTTAA
Y I T H R E S L G T I N T A F K H M K S G D C I R C V L D M K A Stop

gccttttctgaacaaaagtgtgtacttgttctcgactggatgctgtgaactatggagtgtgtcttaaggaggtcttggggttggagg –3′

Legend

Fig. S1 Structure of the *fldA* gene

The nucleotide sequence and deduced amino acid sequence of the *fldA* gene are shown. The underlined sequences indicate introns 1, 2, 3, 4, and 5. The double underlined sequence indicates the initiation codon of FldA.
